# Approaching threat modulates visuotactile interactions in peripersonal space

**DOI:** 10.1007/s00221-016-4571-2

**Published:** 2016-02-19

**Authors:** Alyanne M. de Haan, Miranda Smit, Stefan Van der Stigchel, H. Chris Dijkerman

**Affiliations:** Experimental Psychology, Helmholtz Institute, Utrecht University, Heidelberglaan 1, 3584 CS Utrecht, The Netherlands

**Keywords:** Peripersonal space, Near space defensive, Approaching threat, Visuotactile processing, Visuotactile prediction

## Abstract

The region surrounding our body (i.e. peripersonal space) is coded in a multimodal representation by fronto-parietal bimodal neurons integrating tactile stimuli on the body with nearby visual stimuli. This has often been suggested to serve a defensive purpose, which we propose could be mediated through visuotactile predictions. An approaching threat would then be of particular interest to peripersonal space processing. To investigate this, we asked participants to respond as fast as possible to a tactile stimulus on the hand, while looking at an animation of an approaching or receding spider or butterfly. Tactile stimulation was applied at one of 25 possible time points during the animation. Tactile reaction times were faster when an approaching stimulus was closer to the hand at the time of tactile presentation. Critically, this effect of distance on reaction times was larger when participants saw an approaching spider compared to an approaching butterfly, but only for participants who were afraid of spiders. This finding demonstrates that the perceived threat of an approaching stimulus modulates visuotactile interactions in peripersonal space and is consistent with the idea that visuotactile predictions are important for defensive purposes and maintaining bodily integrity.

## Introduction

We are not alone in this world. Often, the space around us is filled with other animals and objects, and we frequently interact with them. However, not everything around us is innocuous. By predicting the probable consequences of contact with nearby animals, people and objects, we can prepare an appropriate response; for instance, hug a friend or avoid an approaching snowball. Appropriate responses are particularly important in the region directly surrounding your body (e.g. peripersonal space). Evidence suggests that the peripersonal space is represented by fronto-parietal bimodal neurons integrating tactile stimuli on the body with nearby visual (and auditory) stimuli (Rizzolatti et al. 1981). Interestingly, electrical stimulation of peripersonal space neurons in macaque monkeys triggers a pattern of arm movements that is compatible with defence and avoidance behaviour (Cooke and Graziano 2003; Graziano and Cooke 2006). Therefore, it has been suggested that peripersonal space acts as a defence zone or margin of safety around the body (Graziano and Cooke 2006; Sambo et al. 2012). In line with this idea, the size of an individual’s peripersonal space is correlated with trait anxiety (Sambo and Iannetti 2013), with a larger peripersonal space in more anxious individuals.

For defensive purposes, it is relevant to identify nearby objects. Identification will allow predictions of the harmfulness of bodily contact with the object. Touching a cactus will have very different consequences than touching a flower. Consequently, in the light of a defensive purpose of peripersonal space, the implied threat of an object to a certain body part would be expected to have a large influence on tactile processing on that body part. Indeed, threat enhances visuotactile cueing in peripersonal space. In an experiment by Poliakoff et al. (2007), participants were required to make judgements about the vibration frequency of a tactile stimulus (fast or slow) on either the left or the right hand, which was preceded by a visual stimulus near one of the hands. Any visual cue near the hand facilitates tactile discrimination on that hand (see for instance Reed et al. 2006; Tseng and Bridgeman 2011), but Poliakoff et al. (2007) showed that this facilitation was larger when stimuli consisted of threatening pictures rather than neutral pictures.

Most studies on the crossmodal effects of visual threat in peripersonal space have used static images (Lipp and Derakshan 2005; Poliakoff et al. 2007; Van Damme et al. 2009; Brown et al. 2010; Shi et al. 2012). However, an approaching threat is far more relevant and would imply rather different visuotactile predictions than a static one. Moreover, the relevance of a visual threat for visuotactile predictions should increase as it approaches the body. Peripersonal space neurons in macaque monkeys show an increased firing rate if the visual stimulus is moving in the direction of the tactile receptive field (Graziano and Cooke 2006) and tactile judgments are faster at the expected time and location of impact of an approaching visual stimulus (Gray and Tan 2002; Kandula et al. 2015; Cléry et al. 2015). Furthermore, it has been shown that an approaching auditory stimulus facilitates tactile perception when it is perceived within the peripersonal space (Canzoneri et al. 2012). In line with the hypothesised importance of visuotactile predictions, Van Damme et al. (2009) showed that the facilitating effect of visual threat on tactile attention is larger with physical threat pictures (for instance a snake or a knife) than with more general threat pictures (for instance an exploding jet or an angry face) or neutral pictures, suggesting that the effects are related to the predicted consequences of touching the object or animal. In an interesting experiment by Taffou and Viaud-Delmon (2014), participants who were (not) afraid of dogs had to detect tactile stimuli to the hand while a threatening (dogs barking) or non-threatening sound (sheep bleating) was presented from behind the participant. The sounds changed in volume and other aspects such as frequency spectrum and inter-aural differences over the course of 3 s, to mimic an approaching movement. Responses were faster when threatening sounds appeared closer, and this effect depended on the reported fear of dogs. For dog-fearful participants, the sound of barking dogs started influencing tactile detection earlier in the trial than for non-fearful participants. A very similar study by Ferri et al. (2015) also increased sound volume to mimic an approaching movement. They found the same effect as Taffou and Viaud-Delmon (2014): noise sounds that elicited a negative emotion and negative ecological sounds (recording of a screaming woman) started influencing tactile reaction times earlier in the trial than sounds with a neutral or positive valence. This has been interpreted as reflecting a larger peripersonal space when a threat is approaching. These studies, however, did not include a condition in which the sounds mimicked a receding movement, which makes it uncertain whether the reported effects were due to, for instance, changes in stimulus intensity or duration, or to the perception that a threat was approaching.

The aforementioned findings are consistent with the idea that peripersonal space has a defensive purpose (Sambo et al. 2012; Sambo and Iannetti 2013; De Vignemont and Iannetti 2015). Most studies on peripersonal space, however, emphasize the strong link between visual and tactile perception, especially when a visual stimulus is approaching the body (Graziano and Cooke 2006; Huang et al. 2012). If the increased multimodal integration in peripersonal space indeed serves a defensive purpose, mediated through visuotactile predictions, you would specifically expect a large influence of an approaching threat on visuotactile interactions. Surprisingly, while some effects of an approaching visual threat on perceptual and cognitive tasks have been studied (e.g. Vagnoni et al. 2012; Anelli et al. 2013; Witt and Sugovic 2013; Sagliano et al. 2014), the effect on visuotactile interactions in peripersonal space has not yet been investigated. To that purpose, we used a tactile detection task in which participants were asked to respond as fast as possible to a tactile stimulus, while looking at an animation of an approaching or receding spider or butterfly. The animations were presented on a large horizontal flat screen monitor, so they were actually approaching and receding from the participant. To our knowledge, this is the first study investigating approaching threat on visuotactile processing in peripersonal space using realistic stimuli, while controlling for retinal size, duration and location by adding a receding stimulus condition.

## Materials and methods

### Participants

Twenty-six undergraduate and graduate students (1 male, mean age 21 ± 1.1 years) participated in this study. They could receive course credits as a compensation for their time. They were naïve to the purpose of the study, and written informed consent was obtained from all individual participants prior to the experiment. All participants were right handed by self-report. This study was conducted in accordance with the standards of the local ethical committee and the declaration of Helsinki.

### Experimental set-up and stimuli

**Fig. 1:**
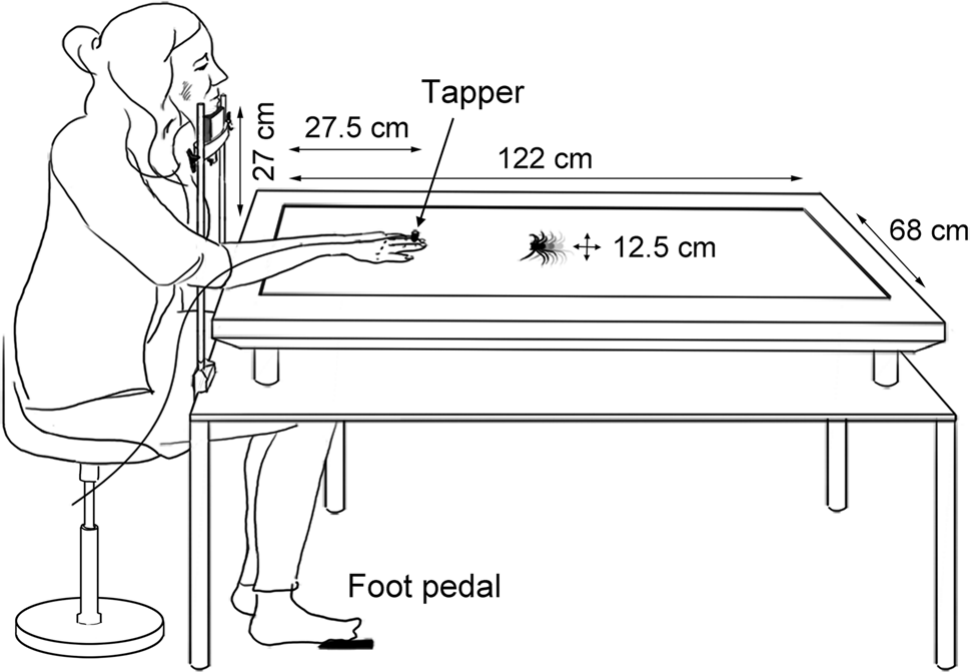
Overview of the experimental set-up. Dimensions are given in the drawing, as well as the positions of the hand, chin rest and foot pedal

Participants were seated in a dark room on the short end of a large flat screen monitor (Philips BDT5530EM/06, screen dimensions 122 × 68 cm), which was placed flat on a table (see Fig. 1). Their heads were stabilised with a chinrest (height 27 cm from the table), and the toes of the right foot were placed on a foot pedal below the table. Participants placed their right hand on the monitor, dorsal side up, with the tip of the middle finger at a distance of 27.5 cm from the edge of the screen.

**Fig. 2:**
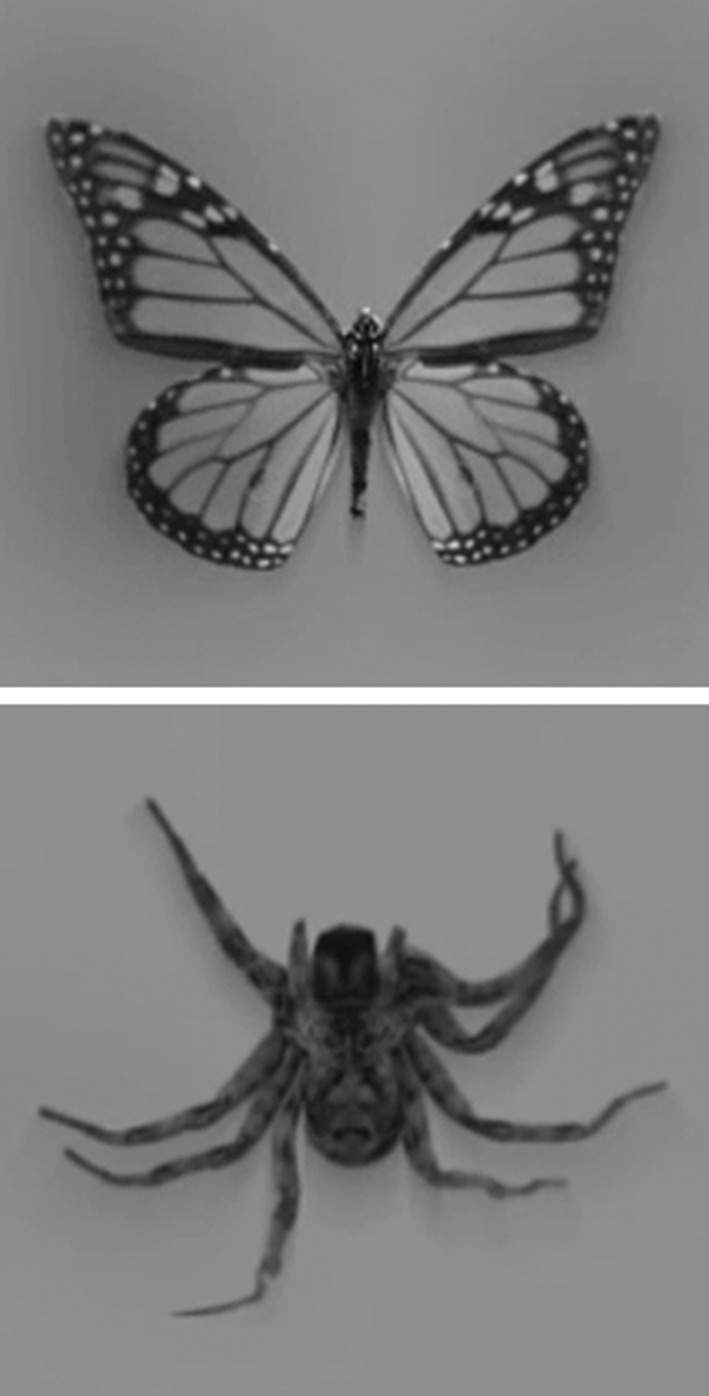
Screenshots of the animations of butterflies and spiders used in the experiment

During each trial, an animation was presented on the monitor (see Fig. 1). Animations consisted of a crawling spider or flying butterfly (see Fig. 2) with a diameter of 12.5 cm, which moved either towards or away from the participants hand in a straight line (start and end locations at 29 and 112.5 cm from the edge of the table, covering 83.5 cm in 4 s or 240 frames at a refresh rate of 60 Hz). The animations were presented on a grey background. Half of the presented animated animals was presented in greyscale, and the other half of the animations were coloured with an orange/yellow colour, to prevent habituation to the stimuli.

At 25 different time points during a trial, the participant could receive a tactile stimulus on their right hand. The first possibility was 560 ms after the start of the animation, corresponding to 15.3 or 71.2 cm from the hand depending on the direction of movement. Then, a tactile stimulus could be applied every 120 ms (but only once per trial), corresponding to once every 2.5 cm. Tactile stimuli consisted of clearly perceivable ‘taps’ with plastic pins with a diameter of 2 mm and were applied using a computer-controlled miniature solenoid tapper (MSTC3 M&E Solve, Rochester, UK) that was attached to the dorsal side of the first phalanx of the middle finger with medical tape. All taps had duration of 10 ms.

### Procedure

After receiving instructions and signing the consent forms, participants were instructed to press the foot pedal with the toes of their right foot (without shoe) as fast as possible when they felt the tactile stimulus, and to keep looking at the animations. Each participant completed 10 practice trials, followed by 4 blocks of 100 trials. Each trial had a duration of 4 s, followed by a 500 ms inter-trial interval in which a black screen was presented. Trials were presented in randomised order within blocks. Each unique movement direction (2) × animal (2) × location (25)—combination was presented 4 times.

### Questionnaires

To clarify for which participants the animation of the crawling spider actually imposed a perceived threat, after the tactile detection task all participants completed the Fear of Spiders Questionnaire (FSQ, Szymanski and O’Donohue 1995) and a second questionnaire in which we used the same questions, but replaced all instances of ‘spider’ with ‘butterfly’ (Fear of Butterflies Questionnaire: FBQ). The FSQ consists of 18 items such as ‘If I saw a spider now, I would think it will harm me.’ which participants had to rate on a 7-point Likert scale (0 = strongly disagree, 6 = strongly agree). Scores on the 18 items are summed, and on the possible 0–108 score range, ratings over 15 points are considered indicative of at least a moderate fear of spiders (Cochrane et al. 2008) or in case of the FBQ, butterflies. Scores on the FSQ were used to divide the group in two. Average scores on the FSQ were 21.6 ± 17.7 (range 2–55), and based on the cut-off score of 15 (Cochrane et al. 2008), half (13) of our participants were at least moderately afraid of spiders (high fear) and the other 13 were not (low fear). According to this score threshold, none of the participants was afraid for butterflies (average score FBQ 3.5 ± 4.0, range 0–14).

### Data analysis

The reaction time to the tactile stimulus was recorded in every trial. Trials with reaction times longer or shorter than the median of all reaction times of a participant ±3 times the median absolute deviation were excluded from further analysis (3.48 %), as suggested by Leys et al. (2013).

Then, the average median tactile reaction times over all 25 time points for each of the four Movement Direction (2) × Animal (2) conditions for the two Fear Groups (2) were analysed with a 2 × 2 × 2 mixed-design ANOVA, followed by separate Movement Direction (2) × Animal (2) repeated measures ANOVA’s for the high- and low-fear groups.

Furthermore, to investigate the influence of location of the visual stimulus on tactile reaction times, we calculated the median reaction time per condition [Movement Direction (2) × Animal (2)] at each time point (25) per participant. For each participant, a linear function was fitted to these median tactile reaction times for each of the conditions using the MATLAB ezyfit toolbox. Linear functions were described by: *y* = *ax* + *b* where ‘*y*’ is the reaction time in ms, *x* is the duration of the animation in ms at the time of the tactile stimulus, ‘*a*’ represents the slope of the linear function and ‘*b*’ the *y*-axis intercept at *x* = 0. We used the slope of the fitted function as a measure of the influence of time point on reaction times, as a steeper slope reflects a higher influence. The *y*-axis intercept gives a measure of the reaction time shortly after the onset of the animation. As the animations moved with a constant speed, the distance of the animation increases linearly with the duration of the animation with receding stimuli, and decreases with approaching stimuli.

The slope parameters and *y*-axis intercepts of the functions with approaching and receding spiders and butterflies for each participant were analysed using Movement Direction (approaching vs. receding) (2) × Animal (butterfly vs. spider) (2) × Fear Group (low vs. high fear) (2) mixed-design ANOVA’s, followed by separate Movement Direction (2) × Animal (2) repeated measures ANOVA’s for the high- and low-fear groups. All further comparisons were made with Bonferroni-corrected paired samples *t* tests. The relation between reported fear of spiders and the effect of perceived threat on slope parameters and *y*-axis intercepts was further investigated using a correlation analysis.

## Results

### Average median reaction times for high- and low-fear participants

A Movement Direction (2) × Animal (2) × Fear Group (2) mixed-design ANOVA on the average median tactile reaction times over all 25 locations showed a trend for a three way interaction [*F*(1,24) = 4.07, *p* = .055, *η*_*p*_^2^ = .145]. Separate Movement Direction (2) × Animal (2) repeated measures ANOVA’s for both fear groups showed no significant main effects or interactions.

### Slope parameters for high- and low-fear participants

**Fig. 3:**
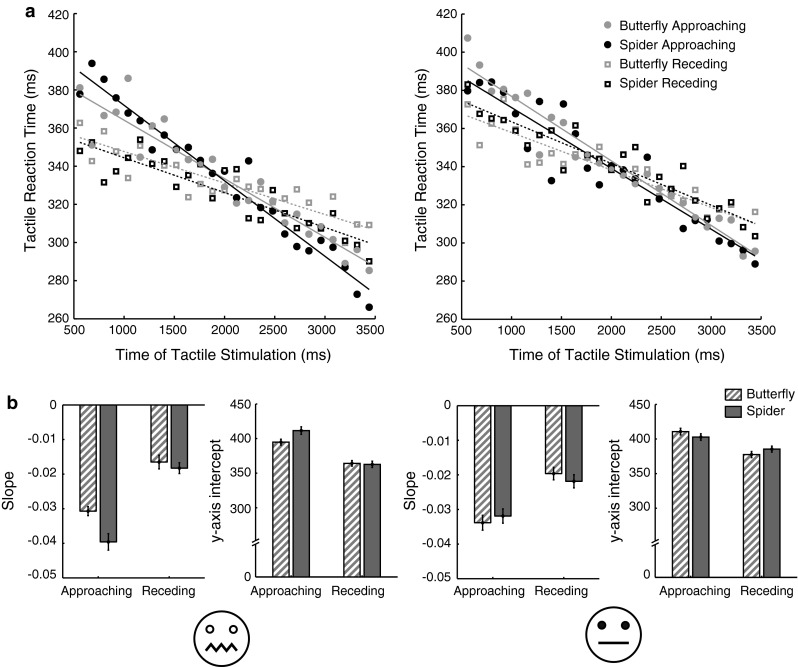
Average median reaction times and fitted linear functions. The average median reaction times to approaching and receding spiders and butterflies at all 25 time points for high- (*left*) and low-fear (*right*) participants, as well as their fitted linear functions (**a**) and average slope and intercept parameters for the fitted linear functions (**b**). Please note that for approaching animations, earlier time points correspond to the stimulus being further way. The *error bars* represent the within-subject standard deviation

Figure 3 shows the average reaction times to approaching and receding spiders and butterflies at all 25 time points for high- and low-fear participants, as well as fitted linear functions. Please note that for approaching animations, earlier time points correspond to the stimulus being further way, while the reverse is true for receding visual stimuli. As can be seen, all slopes are negative, meaning that reaction times decrease when the time between onset of the animation and the tactile stimulus increases. This temporal preparation effect is quite common in reaction time experiments and is due to the onset of the visual stimulus acting as a warning to prepare for an upcoming response (Requin et al. 1991).

A Movement Direction (2) × Animal (2) × Fear Group (2) mixed-design ANOVA showed a main effect of movement direction [*F*(1,24) = 90.15, *p* < .001, *η*_*p*_^2^ = 0.790] and animal [*F*(1,24) = 5.28, *p* = .031, *η*_*p*_^2^ = 0.180] on the slope parameters, but no effect of fear group [*F*(1,24) = 0.02, *p* = .884]. Furthermore, there was no interaction between direction and animal [*F*(1,24) = .33, *p* > .570] but there was an interaction between fear group and animal [*F*(1,24) = 4.74, *p* = .040, *η*_*p*_^2^ = 0.165] and a trend for an interaction between fear group and direction [*F*(1,24) = 3.20, *p* = .086, *η*_*p*_^2^ = 0.118] and a significant three way interaction [*F*(1,24) = 4.80, *p* = .039, *η*_*p*_^2^ = 0.167].

To explore this three-way interaction, separate Movement Direction (2) × Animal (2) repeated measures ANOVA’s were performed for each fear group.

For low-fear participants, there was a main effect of movement direction on the slope parameter [*F*(1,12) = 25.92, *p* < .001, *η*_*p*_^2^ = .684], with steeper slopes when the animations were approaching rather than receding from the hand, but no main effect of animal [*F*(1,12) = 0.01, *p* = .920] and no significant interaction [*F*(1,12) = 1.05, *p* = .326].

For high-fear participants, there was a main effect of movement direction on the slope parameters [*F*(1,12) = 74.47, *p* < .001, *η*_*p*_^2^ = .861], a main effect of animal on the slope parameters [*F*(1,12) = 7.82, *p* = .016, *η*_*p*_^2^ = .395] and an interaction between these factors [*F*(1,12) = 5.05, *p* = .044, *η*_*p*_^2^ = .296]. Paired samples *t* tests (Bonferroni corrected, *α* = .025) showed that slopes were steeper with the spider animation than with the butterfly animation when animations were *approaching* the hand [*p* = .006, *t*(12) = 3.37, *d* = .93] (spider: mean −0.040, 95 % CI [−0.048, −0.031], butterfly: mean −0.031, 95 % CI [−0.038, −0.024]), but not when they were *receding* from the hand [*p* = .458, *t*(12) = 0.767] (spider: mean −0.018, 95 % CI [−0.025, −0.012], butterfly: mean −0.017, 95 % CI [−0.023, −0.011]).

To further investigate the relationship between fear of spiders and the effect of time point on reaction times, we analysed whether the difference in slope between approaching spiders and approaching butterflies correlated with the score on the FSQ in all participants (slope with butterfly–slope with spider, so since all slopes are negative, a positive difference reflects a steeper slope in the spider condition). Indeed, there was a positive correlation between the difference in slope and the FSQ score (Pearson product-moment correlation, *r* = 0.554, *n* = 26, *p* = 0.003). A higher reported fear of spiders was correlated with a larger difference in slope, e.g. a larger effect of time point on reaction times with approaching spiders than with butterflies. So the higher the reported fear of spiders, the more the distance from the animated spider influenced tactile reaction times as compared to with butterflies.

### *y*-Intercept parameters for high- and low-fear participants

The *y*-intercepts of the fitted linear functions for high- and low-fear participants, with approaching and receding spiders and butterflies are depicted in Fig. 3. A Movement Direction (2) × Animal (2) × Fear Group (2) mixed-design ANOVA showed a main effect of movement direction [*F*(1,24) = 66.72, *p* < .001, *η*_*p*_^2^ = .735] on the *y*-intercept parameters, but no effect of animal [*F*(1,24) = 1.81, *p* = .191] and fear group [*F*(1,24) = 0.27, *p* = .608] and no interaction between fear group and animal [*F*(1,24) = 1.81, *p* = .191] or between movement direction and animal [*F*(1,24) = 0.05, *p* = .829]. However, there was a trend for an interaction between fear group and movement direction [*F*(1,24) = 3.27, *p* = .083, *η*_*p*_^2^ = .120] and a significant three way interaction [*F*(1,24) = 8.76, *p* = .007, *η*_*p*_^2^ = .267].

To explore this three-way interaction, separate Movement Direction (2) × Animal (2) repeated measures ANOVA’s were performed.

For low-fear participants, there was a main effect of movement direction on the *y*-intercept parameters [*F*(1,12) = 17.69, *p* = .001, *η*_*p*_^2^ = .596] with higher *y*-intercepts with approaching stimuli, but no main effect of animal [*F*(1,12) < 0.01, *p* = .999] and a trend for an interaction [*F*(1,12) = 3.78, *p* = .076, *η*_*p*_^2^ = .239]. Paired samples *t* tests (Bonferroni corrected, *α* = .025) showed no effect of animal with approaching (*p* = .197) or with receding stimuli (*p* = .120).

For high-fear participants, there was a main effect of movement direction on the *y*-intercept parameters [*F*(1,12) = 58.12, *p* < .001, *η*_*p*_^2^ = .829], again with higher *y*-intercepts with approaching stimuli, but no main effect of animal [*F*(1,12) = 2.79, *p* = .121] and a significant interaction [*F*(1,12) = 5.02, *p* = .045, *η*_*p*_^2^ = .295]. Paired samples *t* tests (Bonferroni corrected, *α* = .025) showed no effect of animal with receding stimuli (*p* = .784) and a trend for an effect of animal with approaching stimuli [*p* = .030, *t*(12) = −2.47].

To further investigate this trend, a Pearson product-moment correlation was run to analyse whether the difference in *y*-intercept with approaching spiders compared to approaching butterflies correlated with the score on the FSQ in all participants (intercept with spider–intercept with butterfly, so a positive difference reflects a higher intercept in the spider condition). There was a positive correlation between the difference in *y*-intercept and the FSQ score (*r* = 0.566, *n* = 26, *p* = 0.003). A higher reported fear of spiders was correlated with a larger positive difference in *y*-intercept, which reflects slower reaction times shortly after onset of a stimulus (corresponding to a large distance from the hand) with approaching spiders as compared to with approaching butterflies.

### The influence of perceived threat in the first and second half of the experiment

During the course of the experiment, the influence of the perceived threat of the animations may change. For instance, the relevance of a spider animation in far space may increase over trial, as participants learn that it will always walk towards their hand. To investigate this, we analysed whether responses in the first half of the experiment differed from those in the second half by introducing Experiment Half as a variable in the repeated measures analysis.

We performed two Experiment Half (2) × Movement Direction (2) × Animal (2) × Fear Group (2) mixed-design ANOVA’s, one for the slope parameters and one for the *y*-intercepts. They showed no main effect of Experiment Half on the slope parameters [*F*(1,24) = 1.757, *p* = .198, *η*_*p*_^2^ = 0.068] and no interactions between Experiment Half and any of the other variables (all *F* < 2.310, *p* > .14). However, for the *y*-intercepts there was a main effect of Experiment Half [*F*(1,24) = 5.026, *p* = .034, *η*_*p*_^2^ = 0.173] with higher *y*-intercepts (i.e. slower reactions at early time points) in the second half of the experiment (on average 400 vs. 387 ms) (and no interactions between Experiment Half and any of the other variables, all *F* < 2.155, *p* > .15). This suggests that while participants got slightly slower in general in the second half of the experiment, the effect of the distance of the stimulus (which is measured by the slope) did not change. There was no interaction between Experiment Half and either movement direction, Animal or Fear Group, so this increase in reaction times seems to be uncorrelated to the visual stimuli. Therefore, there is no indication of a learning or habituation effect present in our data.

## Discussion

The current experiment investigated the influence of an approaching visual threat on visuotactile interactions in peripersonal space with a tactile detection task. Reaction times to tactile stimuli at different time points during the trial were described by fitted linear functions. We hypothesised that when an approaching stimulus is perceived as threatening, its distance from the observer is of more importance to visuotactile predictions than with a neutral stimulus. This would be reflected by steeper slopes of the fitted functions, as the slope reflects the influence of time point/distance on reaction times. (For approaching animations, earlier time points correspond to the stimulus being further way, while the reverse is true for receding visual stimuli.)

The results showed an increase in slope for approaching stimuli compared to receding stimuli. This suggests that the distance of an observer from a visual stimulus has a larger influence on tactile processing when the visual stimulus is approaching. This is in line with our expectations, as the distance from an approaching object is more relevant for visuotactile interactions than from a receding one. Previous studies have shown that peripersonal space processing is indeed particularly influenced by approaching stimuli. For instance, Canzoneri et al. (2012) found that the effect of an auditory moving stimulus on tactile processing of stimuli on the hand was stronger when the auditory stimulus appeared to be approaching. Using visual stimuli, it has been shown that a looming visual stimulus speeds up tactile processing on the face (Cléry et al. 2015). The current experiment shows that this effect depends on the distance to the visual stimulus at the time of the tactile stimulus.

Critically, next to the increase in slope with approaching visual stimuli, our results show an additional increase in slope when participants saw an approaching spider compared to an approaching butterfly, but only for participants that were relatively afraid of spiders. The difference between threatening and non-threatening stimuli was not found when the stimuli were receding from, rather than approaching the stimulated hand, indicating that the effect was distance-dependant, and not caused by either the time of the tactile stimulus within the trial, a difference in temporal preparation effect or the retinal size of the visual stimulus. Additionally, this difference was not found for participants who were less afraid of spiders, which suggests that the stronger influence of distance of the approaching spider animation on reaction times was related to the perceived threat of the stimulus rather than to low-level stimulus features. To summarise, when a perceived visual threat was approaching the body, the distance from this threat influenced tactile processing more than when it was receding from the body. This finding underlines the importance of visuotactile predictions in peripersonal space processing and shows the hypothesised importance of perceived threat in this process.

The current results are consistent with the earlier finding that fear of an auditory stimulus in peripersonal space influenced tactile processing (Taffou and Viaud-Delmon 2014). This study (and many others) focussed on the size of peripersonal space, which is considered to be flexible. An extension of peripersonal space has been shown by various manipulations including tool use (Farnè and Làdavas 2000; Holmes et al. 2004; Ladavas and Serino 2008; Bassolino et al. 2010), artificial body parts (Farnè et al. 2000; Zopf et al. 2010) and mirror images (Maravita et al. 2002; Sambo and Forster 2011). Both auditory threat (Taffou and Viaud-Delmon 2014) and higher trait anxiety (Sambo and Iannetti 2013) result in a larger peripersonal space. However, studies on the reachability of objects showed that threatening stimuli are perceived as not reachable at a closer distance, which would imply a reduction of peripersonal space (Coello et al. 2012; Valdés-Conroy et al. 2012).

The current study sheds a different light on the effect of threat on peripersonal space size. The results showed that the distance from a visual stimulus has a stronger influence on tactile reaction times if it is perceived as threatening, which indicates that the distance to a threatening visual stimulus is more important for visuotactile interaction than to a non-threatening one. While this outcome is similar to findings with approaching auditory stimuli (Taffou and Viaud-Delmon 2014; Ferri et al. 2015), a difference is that they generally find a nonlinear relation between distance and response times while we found a linear effect. As we used a higher measuring resolution (more time points) than previous studies, it is rather unlikely that a nonlinear relation was present in our data, but was missed due to noise. So our data does not suggest a clear distinction between a near and a far region in which the visual threat, respectively, had a large or small influence on tactile processing with a non-continuous transition between them, which has generally been interpreted as a ‘border of peripersonal space’. However, the difference in stimulus modality may be relevant here. As sound localisation is considerably more difficult than visual localisation (e.g. Frens et al. 1995; Alais and Burr 2004; Bowen et al. 2011), using auditory stimuli introduces more uncertainty about the distance and movement of a stimulus. This may have triggered observers to regard a certain area of space around them as a ‘yes, now it is definitely close to me’ (or in other words: relevant) area, with less distinction within this space as the exact location of the auditory stimulus is uncertain. Moreover, for the duration of the current experiment, the monitor was the relevant part of space with respect to visual stimuli. The trajectory of the animations was highly predictable, and the participants knew a spider would always move to the hand when it appeared at the far edge of the monitor. Within this area, the relevance of a spider would increase as it approaches the hand. However, it would be unlikely that it would take a sudden leap in relevance when it crosses the ~70 cm line that is reachable space, where a boundary of peripersonal space is often considered to be (e.g. Iriki et al. 1996; Previc 1998; Witt et al. 2005). This finding suggests that the peripersonal space is not necessarily linked to the space just surrounding the body or extensions of the body such as tools (see also Holmes 2012), but could instead reflect a relevance area in which, depending on the task at hand, objects are for example expected to predict tactile consequences, or in other paradigms, a relevance area in which objects can be manipulated (action space). Again depending on the task this area could have a clear border (for instance between reachable vs. not reachable space) or be reflected by a gradual change in the importance of the distance of objects from the body for multimodal processing, as in the current experiment. This idea is supported by studies showing that peripersonal space is actually not necessarily always in direct connection to the body, but can move to for instance the tip of a hand-held tool (Holmes et al. 2004) or in far space via mirror images (Sambo and Forster 2011) or a shadow (Pavani and Castiello 2004) and can even include parts of other people’s peripersonal space (Maister et al. 2015).

It could be argued that our results were due to differences between the two fear groups in trait anxiety (Sambo and Iannetti 2013) or general arousal (Brendel et al. 2014). A previous study by Sambo and Iannetti (2013) showed that multimodal processing of neutral stimuli close to the face correlated with trait anxiety. If our effects had been due to a higher trait anxiety in the spider-fearful group, the reaction times in the spider-fearful group would have been shorter in general. Instead, the effects clearly were related to the visual stimuli. There was no difference in overall reaction times between the two fear groups, but specifically a difference in the influence of the combination of identity, distance and movement direction of a stimulus. Similarly, this shows that our finding does not simply reflect effects of general arousal.

In the current study, longer reaction times were seen shortly after onset of an approaching stimulus. This may reflect a distance effect (independent of movement) with slower reaction times when a stimulus was far away. However, reaction times shortly after onset of an approaching stimulus were even longer when the stimulus was threatening as shown by the higher *y*-intercept values. Previous studies have described a facilitation of tactile processing when a threatening picture was presented nearby, leading to faster responses than when it was presented further away (for instance near a different body part) (e.g. Poliakoff et al. 2007; Van Damme et al. 2009). These findings are generally explained in terms of attention: a shift in attention towards the location of a visual threat influences tactile events that follow at or near the location of a preceding visual threat. Threatening stimuli indeed are automatically prioritised in attentional selection (Mulckhuyse and Dalmaijer 2015) and can for instance be detected faster than neutral stimuli (Öhman et al. 2001). This increase in attentional capture has been reported for moving threatening stimuli (Carretié et al. 2009), especially when they are approaching (Sagliano et al. 2014). The same attentional bias holds for moving spiders in spider-fearful individuals (Vrijsen et al. 2009). Furthermore, there is a delay in disengagement of attention from threatening stimuli (Koster et al. 2004; Belopolsky et al. 2011; Massar et al. 2011; Vromen et al. 2014; Mulckhuyse and Dalmaijer 2015; for a crossmodal study see Van Damme et al. 2004) and many studies have shown slower responses in or after the presence of negative emotional non-target stimuli (e.g. Fox et al. 2001; Lipp and Waters 2007; Waters and Lipp 2008; McGlynn et al. 2008; Yamaguchi and Harwood 2015). In the current experiment, participants were asked to do two things: look at the visual stimuli (location variable) and respond to tactile stimuli (located on the hand). We therefore assume that spatial attention shifts in the direction of the visual stimulus at the onset of the trial, followed by a shift to the hand at the onset of the tactile stimulus. With threatening stimuli (i.e. approaching spiders in the high-fear group), disengagement from the visual stimulus takes longer, causing participants in the high-fear group to be slower than the low-fear group. However, as the stimulus moves closer, participants in the high-fear group get faster as the visual stimulus becomes more relevant for visuotactile predictions and tactile processing is facilitated.

The current experiment shows an influence of perceived threat on visuotactile interactions even though the used stimuli were not in reality threatening. We are quite used to watching threatening images on screen, and we are well aware that these will not cause us direct physical harm. Probably, the reported effects would be significantly larger when using real spiders, or perhaps virtual reality. However, virtual animations already affected tactile reaction times, which suggests that (perceived) threat indeed has a large influence on visuotactile predictions. Interestingly, when predicting a possible collision with a visual stimulus, the time-to-collision for threatening stimuli is underestimated (Vagnoni et al. 2012). Conformingly, approaching pictures of spiders following a rather unpredictable route appear to be moving faster than non-threatening stimuli, perhaps to trigger faster reactions (Witt and Sugovic 2013). It would therefore be interesting to investigate the influence of the perceived time-to-collision or the predictability of the trajectory of the stimuli in our paradigm.

The increased visuotactile interaction in peripersonal space has often been suggested to serve a defensive purpose. The current results suggest that this could be mediated through visuotactile predictions. That is, perceiving an approaching visual stimulus as threatening would predict negative consequences of bodily contact with the stimulus, which would result in enhanced tactile processing. Here, we have shown a distance-dependent facilitation of tactile processing when a threatening visual stimulus is approaching, which is consistent with this idea. Future studies should further test this hypothesis by investigating whether this facilitation of tactile processing is specific for tactile input applied at the time and location predicted by the approaching threatening visual stimulus.
